# From home deliveries to health care facilities: establishing a traditional birth attendant referral program in Kenya

**DOI:** 10.1186/s41043-015-0023-z

**Published:** 2015-07-16

**Authors:** Angelo Tomedi, Sophia R. Stroud, Tania Ruiz Maya, Christopher R. Plaman, Mutuku A. Mwanthi

**Affiliations:** 1 School of Medicine, MSC09 5040, 1 University of New Mexico, Albuquerque, NM 87131 USA; 2 Department of Family and Community Medicine, School of Medicine, Albuquerque, NM USA; 3 School of Public Health, University of Nairobi, Nairobi, Kenya

**Keywords:** Skilled birth attendants, Traditional birth attendants, Midwifery, Maternal health, Kenya, Childbirth, Maternal health services

## Abstract

**Objective:**

To assess the effectiveness of a traditional birth attendant (TBA) referral program on increasing the number of deliveries overseen by skilled birth attendants (SBA) in rural Kenyan health facilities before and after the implementation of a free maternity care policy.

**Methods:**

In a rural region of Kenya, TBAs were recruited to educate pregnant women about the importance of delivering in healthcare facilities and were offered a stipend for every pregnant woman whom they brought to the healthcare facility. We evaluated the percentage of prenatal care (PNC) patients who delivered at the intervention site compared with the percentage of PNC patients who delivered at rural control facilities, before and after the referral program was implemented, and before and after the Kenya government implemented a policy of free maternity care. The window period of the study was from July of 2011 through September 2013, with a TBA referral intervention conducted from March to September 2013.

**Results:**

The absolute increases from the pre-intervention period to the TBA referral intervention period in SBA deliveries were 5.7 and 24.0 % in the control and intervention groups, respectively (*p* < 0.001). The absolute increases in SBA delivery rates from the pre-intervention period to the intervention period before the implementation of the free maternity care policy were 4.7 and 17.2 % in the control and intervention groups, respectively (*p* < 0.001). After the policy implementation the absolute increases from pre-intervention to post-intervention were 1.8 and 11.6 % in the control and intervention groups, respectively (*p* < 0.001).

**Conclusion:**

The percentage of SBA deliveries at the intervention health facility significantly increased compared to control health facilities when TBAs educated women about the need to deliver with a SBA and when TBAs received a stipend for bringing women to local health facilities to deliver. Furthermore, this TBA referral program proved to be far more effective in the target region of Kenya than a policy change to provide free obstetric care.

## Background

Modern obstetric care and hygienic childbirth practices have been heralded as the main reasons for the decrease in the neonatal mortality rate during the 20th century [1]. This remarkable decline does not extend to developing countries to the same extent [2], where skilled birth attendants (SBAs) preside over fewer than half of deliveries, and where 60 million births occur outside health facilities each year [3]. The lack of appropriate health services during childbirth is also responsible for high maternal mortality rates with at least 42 % of the annual estimated 352,000 maternal deaths occurring during labor and the first 2 days after birth [4]. A large number of maternal deaths and one million neonate stillbirths could be prevented with intrapartum interventions [5]. Most maternal deaths occur during labor, delivery and the immediate postpartum period. The main direct cause is obstetric hemorrhage accounting for 25 % of maternal deaths, infections (15 %), unsafe abortion (13 %), eclampsia (12 %), and obstructed labor (8 %) [6]. These complications are preventable with adequate medical care during and after delivery. It is therefore logical that measures should be taken to increase the number of women who utilize healthcare facilities and SBAs during childbirth.

Maternal and newborn mortality are frequent in Kenya where a high number of deliveries occur at home, often under the supervision of a traditional birth attendant (TBA) [7]. A TBA is defined by World Health Organization as a person who “assists the mother during childbirth and who initially acquired her skills by delivering babies herself or through an apprenticeship to other TBAs” [7].

It is interesting to note that increased training of TBAs has not been shown to reduce maternal mortality during childbirth. A systematic review by Wilson et al. found that the strategy of training and support of traditional birth attendants reduced perinatal and neonatal deaths but had no significant effect on maternal mortality [8]. A joint statement by the World Health Organization, the International Confederation of Midwives (ICM), and the International Federation of Gynecology and Obstetrics (FIGO) also stated “Research indicates that training of TBAs has not contributed to reduction of maternal mortality” [9]. This has led to a call for a concerted effort to replace TBA-attended home births with a health center intrapartum care strategy [10]. Therefore, it is important to create programs that focus on increasing the number, accessibility, and utilization of SBAs [11].

Although TBAs are not as effective as SBAs at reducing childbirth morbidity and mortality, they continue to play an important role in women’s health. In the 1990’s, WHO, UNICEF, and UNFPA described the role of TBA programs in promoting global reproductive health as: a) enhancing the links between modern health care and communities; and b) increasing the number of births attended by trained birth attendants [12]. The ubiquitous presence and availability of TBAs in many rural regions of Africa provides a potential means for contact of pregnant women with the health care system and early referral to health facilities.

According to the 2008–2009 Kenya Demographic and Health Survey, over 92 % of Kenyan women are seen at least once per pregnancy at an antenatal clinic, but only 44 % of deliveries are attended by SBAs. This rate has only improved by 3 % since the previous demographic study conducted in 2003. Additionally, there are regional discrepancies in care during delivery. For example, data collected in 2003 from Western Province revealed that SBAs delivered only 28 % of births. In contrast, in Central Province, the most urban of all provinces, 70 % of women delivered with a SBA [11]. These figures illustrate the minimal improvement in the rate of deliveries attended by SBAs despite efforts made throughout a 5 year period by the Kenya Ministry of Health.

The available data from 2008 show that deliveries assisted by SBAs in Eastern Province were 42.8 % [13]. In contrast to the number for this large area, the Yatta sub-county of Machakos county in eastern Kenya had a much lower rate, showing a need for an intervention program targeted at increasing the rate of childbirths in health facilities with SBAs [14]. According to a recent annual report, the Yatta District SBA rate is only 7 % [15]. Data collected by Tomedi et al. elucidate the beneficial effect of an intervention program targeted at increasing the rate of childbirths in facilities with SBAs in this rural area of eastern Kenya. The study found that a TBA referral system resulted in a large increase in the rate of deliveries in two healthcare facilities [16].

On June 1, 2013 the Kenyan government abolished all fees for maternity services in public facilities with the expressed purpose of increasing SBA deliveries. The purposes of our study were to assess an expansion of the previously studied TBA referral system to a health facility in Yatta sub-county, and to further determine if the TBA referral program increased the SBA delivery rate beyond the effect of the abolishment of fees for services.

## Materials and methods

We conducted a non-randomized controlled trial to investigate if women’s exposure to TBA referrals and a community education program changed their medical facility/SBA-seeking behavior for delivery. We hypothesized that enlisting the support of TBA referrals would significantly increase the number of women who choose to give birth in a health facility with a SBA present.

The site of the study is in southeastern Kenya, in Yatta sub-county of Machakos County. The region is part of the arid and semi-arid lands of Kenya, populated by the Kamba tribe. Dry land rain-fed agriculture and small-scale animal husbandry are the primary source of livelihood for the non-urban population that is included in this study. Extreme poverty and geographic isolation of the population limit access to health care and contribute to a high child and neonatal mortality. The intervention facility was chosen because of its remarkably low percentage (2.4 %) of deliveries of antenatal care patients occurring in a facility with a SBA [16]. The Yatta sub-county has a female population of 141,075 and a total population of 273,519 [17]. A planning meeting was held with the Kisiiki Health Center staff in which the purpose and methods of the intervention were presented. After gaining the support of the clinical staff, they contacted TBAs known to them and recruited the assistance of the chief and the local community health workers to contact other TBAs who were active in the catchment area of the health center. The TBAs who were contacted were asked to attend a meeting with the investigators to discuss the project and request their participation and consent.

In February 2013, we held a meeting with TBAs from the area surrounding the Kisiiki healthcare facility in Yatta sub-county to recruit them into the TBA referral program in order to expand the work of Tomedi et al. [16]. We met with the TBAs and encouraged them to educate or inform their clients about the potential complications that can occur for the mother and newborn at the time of delivery (with examples solicited from experienced TBAs), and why it is important to deliver at a health facility that can handle the complications or provide transport to a higher level of care (e.g. blood transfusion, C-section). Enrolled TBAs were told that they would be compensated with a per diem of KSH 200 (approximately $2.50 USD) for each pregnant woman that the TBAs referred to a facility for an SBA delivery.

The Kenya government/Ministry of Health has emphasized the importance of health facility deliveries and that TBAs are not legitimate providers of health care (e.g. they are practicing without a license). The TBAs are aware of that, but in this and other meetings have stated that pregnant women continue to come to them requesting their assistance, and they feel an obligation to help. The lead author was told in preliminary meetings with TBAs (before the study was started) that they do not charge a fee for their services, but other community sources (e.g. the chief of some of the villages, local health staff) have said that they are paid by the client’s family, an amount that could range from KSH 200 to 500. Because of the government’s stated position regarding TBAs, they have been reluctant to refer the client when complications occur. The pregnant women, when asked about barriers to facility birth, have expressed a fear that they might deliver while traveling the long distance to a health facility. Therefore, in addition to providing the TBAs with an educational message for them to deliver to their clients, the meeting addressed other concerns and barriers. A Ministry of Health official in charge of maternity services for the district spoke of the important role of the TBAs to educate their clients and improve maternal outcomes. She and the hospital staff welcomed and encouraged the involvement of TBAs (up to the point of delivery). It was recognized that the TBAs would continue to be consulted by many families. They are asked to assess if a woman is in active labor, and if there likely is adequate time for the trip to the health facility. The TBA then accompanies the woman in case delivery should occur en route. The stipend paid to the TBA is considered a “per diem” to defray her personal expenses for the trip to the health facility. The amount of the stipend was chosen because the volunteer community health workers (CHWs) in the target area of the non-governmental organization that supports this project are paid a “per diem” of KSH 200 per day of work. The number of TBAs participating in the referral program was 38.

The number of births from the Kisiiki area, which was estimated by the number of new PNC patients, was compared to the rural control facilities in the Yatta sub-county. Data from the Ministry of Public Health and Sanitation (MOPHS) facilities’ records was used to determine PNC visits and pregnant women who delivered at a rural health facility with or without a TBA.

The control facilities consisted of 28 rural dispensaries and health centers within the Yatta sub-county that did not participate in the TBA referral program. The two intervention facilities for which results were reported in the previous study [16] were excluded from this analysis because we did not have data from them for each time interval. The two urban hospitals were also excluded, since the focus of this study is on rural health facilities. The number of first visits of PNC patients was used to estimate the number of pregnancies because the actual number of pregnancies cannot be determined from the available government statistics. Over 90 % of pregnant women are seen for at least one PNC visit, so the number of first PNC patient visits approximates the number of viable pregnancies. Informed consent was obtained from the participating TBAs.

The pre-intervention data was collected from July 2011 through February 2013. In the control facilities, the number of new PNC patients and the number of deliveries were 2112 and 218 respectively. The intervention facility (Kisiiki) recorded 334 new PNC patients and 12 deliveries during the same time period.

These pre-intervention figures were compared with post intervention data collected from March 1, 2013 through May 31, 2013 (prior to enactment of the policy where maternity services are free at health care facilities) and from June 1, 2013 through September 30, 2013 (after the enactment of the policy). Therefore, the post intervention period was divided into subintervals, one before the free maternity care policy was enacted and one after the policy went into effect. These periods were analyzed separately to assess changes in SBA-attended birth rates related to the effect of the change in the government policy.

### Ethics

The University of New Mexico Human Research Protections Office and the Kenyatta National Hospital-University of Nairobi Ethics and Research Committee in Kenya approved the study.

### Statistics

The SBA rate was calculated as a percentage of the prenatal care (PNC) patients who delivered at the facility, with the denominator being the number of first visit prenatal care patients and the numerator being the number of deliveries at the facility. The total number of the PNC patients and deliveries at all non-intervention rural facilities were used for the control percentages. Chi-square tests were used to test differences in percent of SBA (facility) deliveries between intervention and control groups for individual time periods: baseline rates (pre-intervention), for the rates during the entire 7-month intervention period, and for the rates during the two parts of the intervention period (before and after the implementation of the free maternity care policy). Differential changes over time between the intervention and control groups were tested using binomial regression with a term for the interaction between group and time. Statistical analyses were performed using Stata version 13 (StataCorp LP, College Station, TX, USA).

## Results

During the pre-intervention (baseline) period, the percent of SBA deliveries was significantly higher in the control facilities (10.3 %) than in the intervention facility (3.6 %) (χ^2^ = 15.33; *p* < 0.001) (Table 1).

**Table 1:** Pre intervention percent of PNC patients with SBA deliveries

MOPHS Facility Intervention site			
	Deliveries	New PNC patients	% Deliveries/ PNC
Kisiiki	12	334	3.6
Control Yatta District Facilities			
	Deliveries	New PNC patients	% Deliveries/ PNC
TOTAL	218	2112	10.3
Ikombe	61	181	33.7
Kauthulini	3	104	2.9
Kikesa	25	333	7.5
Kinyaata	15	213	7.0
Kitheuni	27	157	17.2
Kithimani	38	360	10.6
Kwamwatu	5	51	9.8
Kyanzavi	0	3	0.0
Kyasioni	11	150	7.3
Mamba	0	83	0.0
Mbembani	2	34	5.9
Ndalani	7	160	4.4
Nthungululu	23	99	23.2
NYS Mavoloni	1	116	0.9
St. Kizito	0	68	0.0

July 2011- February 2013

**Fig. 1:**
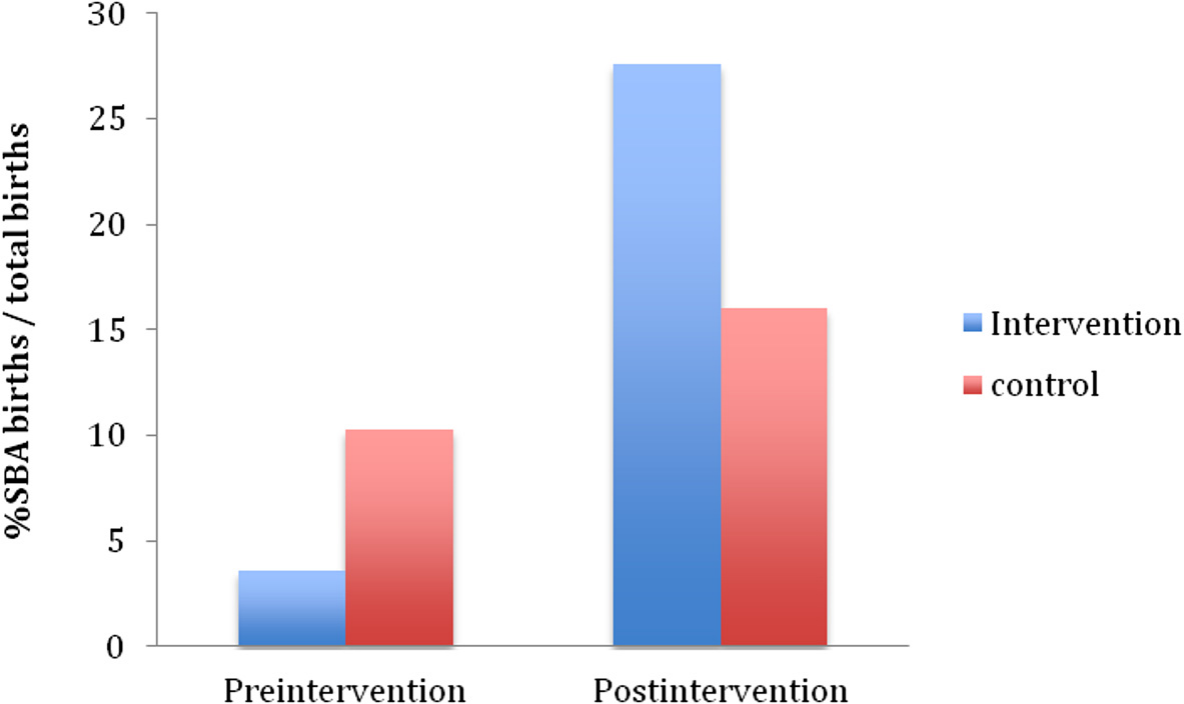
Pre and post intervention percentage of SBA births. 1 Pre-intervention (July 2011–February 2013). 2 Post-intervention (March 2013–September 2013) *p* < 0.001

During the 7-month intervention period, 16.0 % of PNC patients delivered with an SBA at the control facilities, compared to 27.6 % of PNC patients at the intervention facility (Tables 2, 3, Fig. 1). The absolute increases (from pre-intervention to post-intervention) in SBA facility deliveries were 5.7 and 24.0 % in the control and intervention groups, respectively (*z* = 4.21; *p* = 0.001).

**Table 2:** Post-intervention percent of PNC patients with SBA deliveries before free obstetric care policy (i.e. prelegislation)

MOPHS Facility Intervention site			
	Deliveries	New PNC patients	% Deliveries/ PNC
Kisiiki	11	53	20.8
Control Yatta District Facilities			
	Deliveries	New PNC patients	% Deliveries/ PNC
TOTAL	54	359	15.0
Ikombe	5	35	14.3
Kauthulini	2	17	11.8
Kikesa	8	61	13.1
Kinyaata	6	38	15.8
Kitheuni	4	24	16.7
Kithimani	6	62	9.7
Kwamwatu	2	10	20.0
Kyanzavi	0	0	0.0
Kyasioni	5	18	27.8
Mamba	0	12	0.0
Mbembani	3	9	33.3
Ndalani	6	32	18.8
Nthungululu	7	13	53.8
NYS Mavoloni	0	13	0.0
St. Kizito	0	15	0.0

March 2013-May 2013

**Table 3:** Post-intervention Deliveries percent of PNC patients with SBA deliveries after free obstetric care policy (i.e. post legislation)

MOPHS Facility Intervention site			
	Deliveries	New PNC patients	% Deliveries/ PNC
Kisiiki	24	74	32.4
Control Yatta District Facilities			
	Deliveries	New PNC patients	% Deliveries/ PNC
TOTAL	81	483	16.8
Ikombe	8	33	24.2
Kauthulini	3	29	10.3
Kikesa	19	69	27.5
Kinyaata	4	40	10.0
Kitheuni	10	60	16.7
Kithimani	8	86	9.3
Kwamwatu	2	11	18.2
Kyanzavi	0	1	0.0
Kyasioni	5	21	23.8
Mamba	1	21	4.8
Mbembani	5	10	50.0
Ndalani	1	42	2.4
Nthungululu	15	24	62.5
NYS Mavoloni	0	22	0.0
St. Kizito	0	14	0.0

June 2013- September 2013

**Fig. 2:**
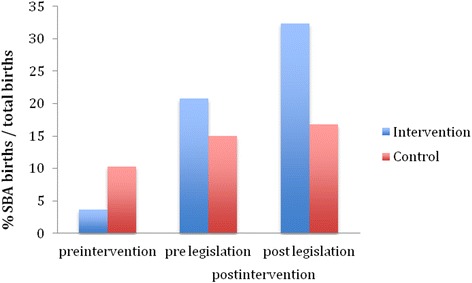
Pre and post legislation percentage of SBA births. 1 Pre-intervention (July 2011-February 2013). 2 Pre-legislation (March 2013–May 2013) *p* < 0.001. 3 Post-legislation (June 2013–September 2013)

The Kenya government implemented a policy that provided free maternity care halfway through our post intervention period. In order to assess the effect of this new policy on SBA deliveries, the 7-month intervention period was divided into two sub- periods (Table 4, Fig. 2):

**Table 4:** Summary of percentage of PNC patients with SBA deliveries

Pre-intervention	12/334 (3.6 %)	218/2112 (10.3 %)	*P* < 0.001
Post-intervention Before free OB care	11/53 (20.8 %)	54/359 (15.0 %)	
Post-intervention After free OB care	24/74 (32.4 %)	81/483 (16.8 %)

1. Before the free maternity care policy (Table 2), 15.0 and 20.8 % of PNC patients had SBA deliveries at the control and intervention sites respectively. The absolute increases in SBA facility deliveries between the baseline period and the first intervention sub-period were 4.7 and 17.2 % in the control and intervention groups, respectively (*χ*^2^ = 2.07; *p* = 0.04).
2. After the free maternity care policy implementation (Table 3), 16.8 and 32.4 % of PNC patients had SBA deliveries at the control and intervention sites respectively (*χ*^2^ = 10.29; *p* < 0.001). The absolute increases in SBA facility deliveries between the baseline period and the period with free maternity care were 1.8 and 11.6 % in the control and intervention groups, respectively (*z* = 3.84; *p* < 0.001).

In the control facilities, the SBA delivery rate increased from 15 % in the first intervention period (before the free maternity care policy) to 16.8 % after the policy implementation (*χ*^2^ = 0.46; *p* = 0.50).

## Discussion

This study shows that a TBA referral and maternal education program was associated with an increase in the percentage of SBA deliveries. We found a significant increase in delivery rates in the before-after comparison at the intervention site, as well as in the comparison of the control and the intervention sites during the study period. The implementation of the Kenyan government policy of free obstetric care at hospitals was enacted with the explicit purpose of increasing the number of women who choose to deliver at health care facilities. However, there was no significant increase in SBA deliveries observed at the control sites, or any further increase in the SBA rate in the intervention facility, after the Kenya government implemented the policy of free maternity care.

Two control facilities were excluded from the statistical analysis of the results. Matuu District Hospital and Matuu Mission Hospital are located in an urban area and had high numbers of PNC patients and deliveries. We attribute these high numbers to the fact that both facilities were located in an area with accessible transportation, increased education level, and increased resources. Because our study aims at targeting rural communities, we determined that these two facilities are not appropriate control sites.

No qualitative or quantitative data were collected that would explain the reason for the increase in SBA deliveries, so the reasons are speculative. Most if not all of the TBAs are aware of potential childbirth complications, though the discussion led by respected health care professionals may have served as a reminder and helped them prepare a message for the pregnant women who consult them. However, the authors speculate, based on informal observations and feedback, that the most important components of the intervention were likely the recognition of their importance in the community and the offer from health care professionals and government officials for them to participate in an important role, at a time when they felt excluded or ostracized. The stipend was also clearly an important component, even though it may have been less than the fee they might have received from the client. Our impression from TBA interactions is that there would be far less participation if they received nothing for their effort and had to pay their own transportation costs to get to the health facility. The stipend can only be sustainable as long as the non-governmental organization (NGO) provides the funds, or the government takes over that responsibility. The amount of funds required is small relative to other costs of the health care system. Other NGOs and low-income developing country governments have decided to pay a salary to lay CHWs for community work, at greater cost than this TBA program. However, the hope is that over time the “norm” in the community will be a health facility delivery rather than a home delivery, and the TBA stipend will not be necessary.

There are recognized limitations of this quasi-experimental study. The relatively brief time period and the number of deliveries in the target facilities limit the power and strength of the conclusions. The intervention and control sites were not randomly assigned, which increases the risk of the influence of potential confounding variables. Unmeasured factors, such as improved accessibility and cultural issues or preferences may have contributed to the results, but are not likely to have influenced the outcome significantly more in the intervention region than in the control region. We did not collect qualitative or quantitative data on the population characteristics, potential confounders, and about the community’s reasons for lack of response to the Kenya policy change. The restriction of the study population to one ethnic group in one district limits the generalizability of the results.

The efforts in Kenya thus far to increase SBA deliveries has met with very limited success. Our approach of involving TBAs in a referral program could offer the potential for Kenya and other sub-Saharan African countries to improve SBA facility delivery rates at a very low cost. A larger study of this approach would seem warranted in other regions and countries where TBAs play an active role in the community.

## Conclusion

The percentage of SBA deliveries at the intervention health facility significantly increased compared to control health facilities when TBAs educated women about the need to deliver with a SBA and when TBAs received a stipend for bringing women to local health facilities to deliver. Furthermore, this TBA referral program proved to be far more effective in the target region of Kenya than a policy change to provide free obstetric care.

## References

[1] Piekkala P, Erkkola R, Kero P, Tenovuo A, Sillanpaa M (1985). Declining perinatal mortality in a region of Finland, 1968–82. Am J Public Health.

[2] Lawn JE, Lee AC, Kinney M, Sibley L, Carlo WA, Paul VK (2009). Two million intrapartum-related stillbirths and neonatal deaths: where, why, and what can be done?. Int J Gynaecol Obstet.

[3] UNICEF: State of the World’s Children 2009 (2008). In maternal and newborn health.

[4] Hogan MC, FK K, Naghavi M, Ahn SY, Wang M, Makela SM (2010). Maternal mortality for 181 countries, 1980–2008: a systematic analysis of progress towards millennium development goal 5. Lancet.

[5] Bhutta ZA YM, Lawn JE. Stillbirths: how much difference can we make and at what cost? Lancet. 2011. 10.1016/S0140-6736(10)62269-6 21496906

[6] Wanjira C, Mwangi M, Mathenge E (2011). Delivery practices and associated factors among mothers seeking child welfare services in selected health facilities in Nyandarua south district, Kenya. BMC Public Health.

[7] WH Organization (1992). Traditional birth attendants: a joint WHO/UNICEF/UNFPA statement.

[8] Wilson A, Gallos ID, Plana N, Lissauer D, Khan KS, Zamora J (2011). Effectiveness of strategies incorporating training and support of traditional birth attendants on perinatal and maternal mortality: meta-analysis. BMJ.

[9] Making pregnancy safer: the critical role of the skilled attendant (2004). A joint statement by WHO, ICM and FIGO.

[10] Campbell OM, Graham WJ (2006). Lancet maternal survival series steering group. Strategies for reducing maternal mortality: getting on with what works. Lancet.

[11] Wilson N. Liambila, Population Council. Nairobi Office, Kenya. Ministry of Health, University of Nairobi. Safe Motherhood Demonstration Project, Western Province: Final Report. Population Council, Sub-Saharan Africa Region, Nairobi Office. 2004:95–9.

[12] United Nations Population Fund: office of oversight and evaluation. Support to traditional birth attendants. 1996. <http://www.unfpa.org/monitoring/pdf/n-issue7.pdf> accessed July 2012.

[13] Kenya National Bureau of Statistics (KNBS) and ICF Macro (2010) Kenya Demographic and Health Survey 2008–2009. Calverton, Maryland: KNBS and ICF Macro; 2010. http://dhsprogram.com/pubs/pdf/fr229/fr229.pdf.

[14] Olenja J, Godia P, Kibaru J, Egondi T. Influence of Provider Training on Quality of Emergency Obstetric Care in Kenya. 2004 Kenya Service Provision Assessment Survey. Kenya Working Papers No.3, DHS Program. http://dhsprogram.com/pubs/pdf/wpk3/wpk3.pdf.

[15] Annual Operational Plan for Yatta District. Consolidated District Health Sector Plan 2011/2012. Republic of Kenya Ministry of Health, 2011. Afya House, Cathedral Road, Nairobi 00100, Kenya.

[16] Tomedi A, Tucker K, Mwanthi MA. A Strategy to Increase Skilled Attendant Births in Kenya. Int J Gynaecol Obstet. 2013;120(2):152-5. 10.1016/j.ijgo.2012.09.013 23195287

[17] Kenya 2009 Census Data. Mars Group Kenya. 2012. http://www.marsgroupkenya.org/census/?data=phoudk&province=4:Eastern+Province&district=423:YATTA+District.

